# The Influence of Metformin to the Transcriptional Activity of the mTOR and FOX3 Genes in Parapancreatic Adipose Tissue of Streptozotocin-Induced Diabetic Rats

**DOI:** 10.25122/jml-2020-0029

**Published:** 2020

**Authors:** Denis Anatolievich Putilin, Sergey Yuryevich Evchenko, Larisa Yaroslavivna Fedoniuk, Olexandr Stepanovich Tokarskyy, Oleksandr Mikhailovich Kamyshny, Liudmyla Mikhailivna Migenko, Serhiy Mikhailovich Andreychyn, Iryna Ihorivna Hanberher, Tetyana Oleksandrivna Bezruk

**Affiliations:** 1. Department of Normal Physiology, Zaporizhzhia State Medical University, Zaporizhzhia, Ukraine; 2. Department of Microbiology, Virology and Immunology, Zaporizhzhia State Medical University, Zaporizhzhia, Ukraine; 3. Medical Biology Department, I. Horbachevsky Ternopil National Medical University, Ternopil, Ukraine; 4. Department of Medical Biochemistry, I. Horbachevsky Ternopil National Medical University, Ternopil, Ukraine; 5. Department of Microbiology, Virology and Immunology, Molecular Genetics Laboratory, Zaporizhzhia State Medical University, Zaporizhzhia, Ukraine; 6. Second Department of Internal Medicine, I. Horbachevsky Ternopil National Medical University, Ternopil, Ukraine; 7. Department of Propedeutics of Internal Medicine and Phthisiology, I. Horbachevsky Ternopil National Medical University, Ternopil, Ukraine; 8. Department of Internal Medicine and Infectious Diseases, Bukovinian State Medical University, Chernivtsi, Ukraine.

**Keywords:** diabetes mellitus, streptozotocin, rat model, adipose tissue, mTOR, Foxp3, IL1β, IL17A

## Abstract

The mammalian target of rapamycin is not only a central regulator of lipid metabolism that controls the processes of adipogenesis and lipolysis but also a regulator of the immunometabolism of immune cells that infiltrate adipose tissue. In turn, the level of progression of diabetes is significantly influenced by the Treg subpopulation, the complexity and heterogeneity of which is confirmed by the detection of numerous tissue-specific Tregs, including the so-called VAT Tregs (visceral adipose tissue CD4+Foxp3+ regulatory T cells). Therefore, the purpose of the study was to determine the mRNA expression levels of mTOR, Foxp3, IL1β, and IL17A genes in rat parapancreatic adipose tissue with experimental streptozotocin-induced diabetes mellitus, with or without metformin administration. The experiments were performed on male Wistar rats with induced diabetes as a result of streptozotocin administration. Molecular genetic studies were performed using real-time reverse transcription-polymerase chain reaction. The development of diabetes caused transcriptional activation of the mammalian target of rapamycin protein kinase gene, as well as increased mRNA expression of the pro-inflammatory cytokines IL1β and IL17A, but did not affect Foxp3 mRNA expression. The intervention with metformin in diabetic rats inhibited the mammalian target of rapamycin mRNA expression and caused an increase in the transcriptional activity of the Foxp3 gene in parapancreatic adipose tissue.

## Introduction

Previous studies showed that the onset of experimental streptozotocin-induced diabetes mellitus (ESIDM) resulted in transcriptional induction of genes for glucose transporters Glut1 and mammalian target of rapamycin (mTOR) protein kinase in cells of pancreatic lymph nodes (PLN), as well as an increased number of TLR2+ and TLR4+ adipocytes and higher density of TLR2+ and TLR4 + receptors on their membrane in parapancreatic tissue [1, 2]. The detected increase in mRNA mTOR protein kinase levels, as well as mTOR protein level in PLN cells under diabetes, may be a trigger for cell differentiation into effector pro-inflammatory subpopulations Th1 and Th17. In addition, adipose tissue (AT) contains well-defined clusters of cells of the innate and adaptive immune system [3], which infiltrate adipocytes and balance the level of pro-inflammatory signaling in AT and production of such cytokines as IL1β, IL17A, TNFα, IFNγ, which are capable of directly affecting the progression of insulitis [4]. In turn, mTOR is an essential regulator of lipid homeostasis [5], as well as immunometabolism of lymphocytes infiltrating AT [6]. Therefore, the purpose of the study was to determine the mRNA expression level of mTOR, Foxp3, IL1β, and IL17A genes in parapancreatic adipose tissue (PPAT) of ESIDM rats after metformin administration.

## Material and Methods

The experiments were performed on 60 male Wistar rats weighing 115-135 grams, which were obtained from the kennel of the Veterinary Medicine Association (Biomodelservice, Kyiv, Ukraine). The animals were randomly divided into five experimental groups, 12 rats per each group: control rats, which were intraperitoneally injected 0.5 ml of 0.1 M citrate buffer (pH = 4.5) (group 1); rats with 3 weeks ESIDM (group 2); rats with 5-week ESIDM (group 3); rats with 3-week ESIDM (group 4) and 5-week ESIDM (group 5) were given metformin intragastrically at a dose of 50 mg/kg daily for 3 and 5 weeks, respectively, starting from day 1 after diabetes induction.

For the induction of ESIDM, streptozotocin (STZ) (Sigma-Aldrich, USA) was dissolved in 0.5 ml of 0.1 M citrate buffer (pH 4.5) and immediately administered intraperitoneally at a dose of 50 mg/kg. The time elapsed from the date of STZ administration was interpreted as the duration of diabetes in the subsequent presentation of the results. Blood glucose concentration in each rat before the sacrifice was measured using the glucose oxidase method (BIONIMERigh testTMGM 110, Switzerland) on the blood samples drawn from the tail vein at 12 hours, and on day 1, 3, 21 and 35 after STZ injection. Glycemia evaluation was carried out after 6 hours since the last meal. The animals were sacrificed on day 21 (groups 2 and 4) and day 35 (groups 3 and 5) after the diabetes induction by decapitation under thiopental anesthesia.

PPAT samples from each rat were removed, immersed in Bouin's fixative solution (15 parts of saturated aqueous picric acid solution, 5 parts of formaldehyde, 1 part of acetic acid) for 20 hours, followed by tissue dehydration using ascending concentrations of ethanol, and then placed in paraffin blocks (Paraplast R, Sigma-Aldrich, USA).

Molecular genetics experiments were conducted on archival material not older than three years. The total RNA was obtained from histological sections with a thickness of 15 μm by their deparaffinization in xylene, followed by rehydration in descending concentrations of ethanol (100%, 96%, 70%). Chemicals for Bouin's solutions, as well as xylene and ethanol, were of laboratory grade and obtained from Sigma-Aldrich, USA. The total RNA was isolated using a Trizol RNA Prep 100 kit (Isogen Lab., LTD, Russian Federation) containing Trizol reagent (a lysing reagent comprising guanidine thiocyanate as denaturing agent and phenol with pH = 4.0) and ExtraGene E (suspension of ion exchange mixture). The RNA was isolated according to the manufacturer's instructions.

Reverse transcription reaction and cDNA synthesis were carried out using a commercial kit OT-1 (Synthol, Russian Federation). The reaction mixture with a total volume of 25 μl contained 1 μl of Random-6 primer, 2 μl of total RNA, 8.5 μl of deionized RNAse-free H2O, 12.5 μl of 2.5× reaction mixture, and 1 μl of MMLV-reverse transcriptase. Reverse transcription was performed at 45°C for 45 minutes, followed by M-MLV-RT heat inactivation (92°C, 5 minutes), as recommended by the manufacturer. CFX96 ™Real-Time PCR Detection Systems Amplifier (Bio-Rad Laboratories, Inc., USA) and Maxima SYBR Green/ROX qPCR MasterMix (2X) reagent kit (ThermoScientific, USA) were used to measure expression levels of mRNAs of the investigated genes, namely, Foxp3 (NM_001108250.1), mTOR (NM_019906.1), IL1β (NM_031512.2), and IL17a (NM_001106897.1). The final reaction mixture for amplification included SYBR Green dye, Maxima HotStartTaq DNA Polymerase, 0.2 μl of each forward and reverse specific primers, 1 μl of the template (cDNA). The reaction mixture was brought to a total volume of 25 μl with the addition of deionized H2O. Forward and reverse specific primer pairs for amplification of the investigated and reference genes were chosen using the PrimerBlast software (www.ncbi.nlm.nih.gov/tools/primer-blast) and manufactured by Metabion, Germany and ThermoScientific, USA (Table 1).

**Table 1:** Specific primer pairs for amplification of the investigated and reference genes.

Gene	Gene ID (NCBI-NLM database)	Primer	Tm,^0^ C	Product length (bp)	Exon junction
Foxp3	NM_001108250.1	F = TCTGGCCAAAAGACAGGTGG R = CTGTCCCAGGGTCCACAAAG	60 60	40	2577/2578
mTOR	NM_019906.1	F = CGAGACTTGGAAGTCAGCCAC R = TCTGAGGCAGGCTGGATAACG	60 61	61	214/215
IL1β	NM_031512.2	F = TCTTTGAAGAAGAGCCCGTCC R = GGTCGTCATCATCCCACGAG	60 60	48	354/355
IL17a	NM_001106897.1	F = CTGGACTCTGAGCCGCAATG R = TGCCTCCCAGATCACAGAAG	61 59	58	297/298
GAP DH		F = GCCTGGAGAAACCTGCCAAG R = GCCTGCTTCACCACCTTCT	61 60	52	825/826

Initial DNA denaturation was carried out for 10 minutes at 95°C, followed by the amplification process consisting of 45 cycles. Each cycle was performed under the following conditions: denaturation - 95°C for 15 sec., annealing - 5961°C for 30-60 sec. (depending on the target gene and primer set), elongation - 72°C for 30 sec. Glyceraldehyde-3-phosphate dehydrogenase gene (GAPDH) was used as a reference gene to determine the relative changes in the expression level of the studied genes and normalize the data. The relative normalized number of cDNA target genes was determined by the ΔΔCt method. Statistical analysis of PCR data was performed using the CFX Manager ™ software (Bio-Rad, USA).

Negative controls were included in all experiments, including the absence of MMLV-reverse transcriptase in cDNA synthesis, absence of mRNA in the reaction of cDNA synthesis, absence of cDNA in the real-time PCR reaction. All amplification reactions were performed on individual samples (one sample from each experimental animal) in three repetitions, resulting in a total of 36 data points in each investigated group.

## Results and Discussion

STZ injection in experimental animals led to the development of a pathological process relevant to diabetes mellitus. Specifically, blood glucose concentration increased to 12.23 ± 0.4 mM and 14.39 ± 0.7 mM in 3-week ESIDM rats and 5-week ESIDM rats, respectively, compared to control rats (3.37 ± 0.08 mM, p <0.05). Additionally, polydipsia, hyperphagia, and polyuria were observed as the main symptoms characteristic for type 1 diabetes mellitus.

**Figure 1:**
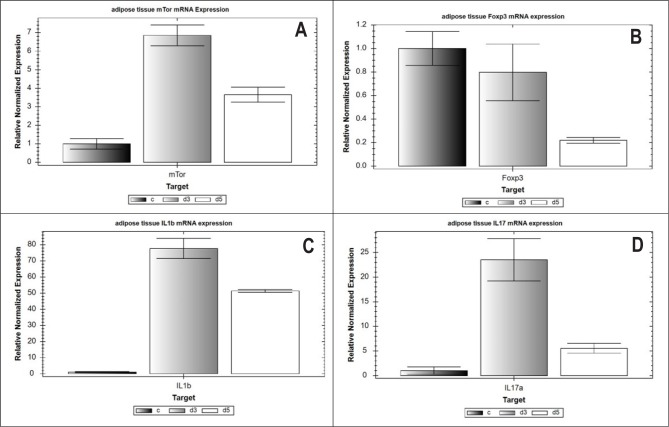
Relative normalized mRNA number of mTOR genes (A), Foxp3 (B), IL1β (C) and IL17A (D) in PPAT. Normalization performed using the ΔΔCt method with GAPDH as the reference gene. C-control, d3, d5 – diabetic rats of 3 and 5 weeks, respectively.

STZ-induced metabolic changes caused transcriptional activation of the mTOR protein kinase gene in PPAT cells. Specifically, STZ-induced diabetes led to an increase in the mTOR mRNA content by 6.8 times (p <0.05) at 3 weeks and by 3.6 times (p <0.05) at 5 weeks after the start of the pathological process compared to control healthy rats (Figure 1A). These changes did not affect the mRNA expression of the transcriptional regulator of differentiation of T-regulatory cells - Foxp3 - in 3-week ESIDM rats (group 2), but decreased it by 4.6 times (p <0.05) in 5-week ESIDM rats (group 3) (Figure 1B) compared to the control group of rats (group 1). As previously shown, increased TLR2+ - and TLR4+ adipocyte number and the density of TLR2+ - and TLR4+ receptors on the membrane of these adipocytes in parapancreatic tissue could possibly induce pro-inflammatory signaling [7]. Thus, the level of IL1β pro-inflammatory cytokine mRNA expression increased by 77.7 times (p <0.05) in 3-week diabetic rats and by 51.3 times (p <0.05) in 5-week diabetic rats (Figure 1C), while Th17-dependent cytokine IL17A mRNA expression increased by 23.5 times (p <0.05) and 5.5-fold (p <0.05), respectively, compared to control intact rats (Figure 1 D).

**Figure 2:**
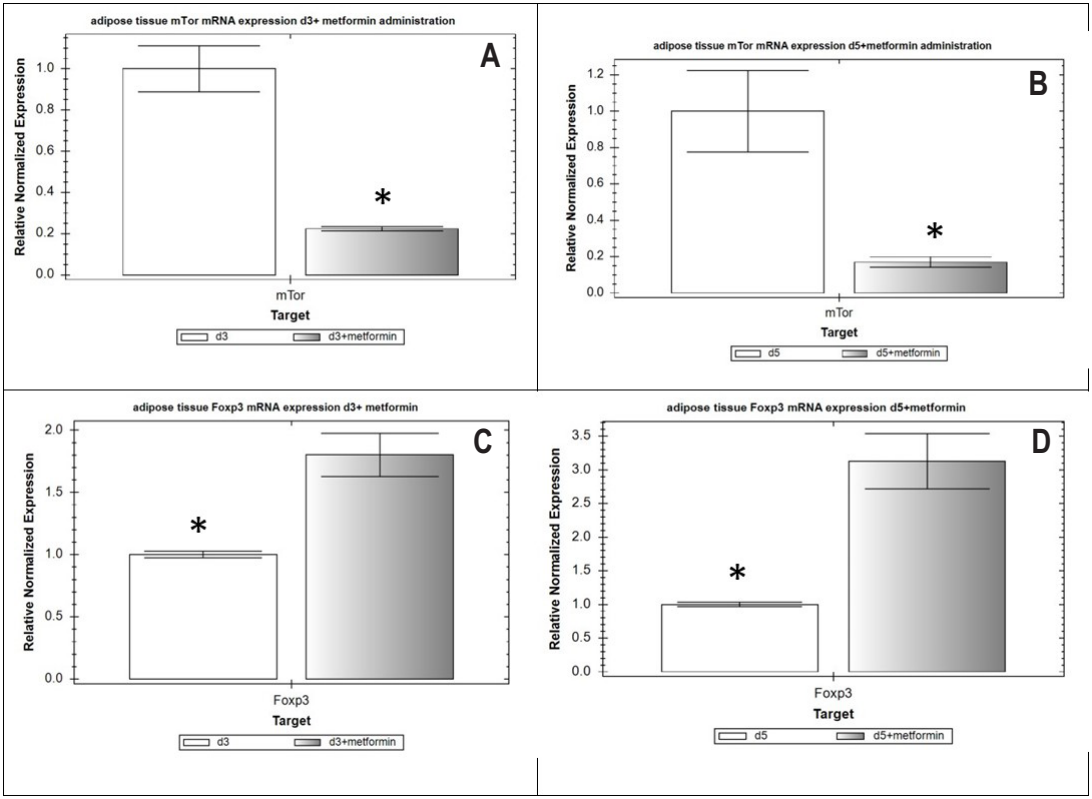
Relative normalized mRNA number of mTOR genes(A-B), Foxp3 (C-D) in PPAT with or without metformin administration. Normalization performed using the ΔΔCt method with GAPDH as the reference gene. d3, d5 - diabetic rats of 3 and 5 weeks, respectively; d3+metformin, d5+metformin - diabetic rats of 3 and 5 weeks, respectively treated with metformin. Asterisk (*) indicates a significant difference (p < 0.05).

Metformin administration inhibited mTOR expression, as a 4.5-fold decrease (3-week diabetic rats, group 4) and 5.9-fold decrease (5-week diabetic rats, group 5) in the mRNA level of the mTOR was observed (p <0.05) in the PPAT compared to untreated ESIDM rats (Figure 2AB). Such transcriptional repression of mTOR led to an increase in the level of transcriptional activity of the Foxp3 gene in Treg cells of PPAT. Thus, the relative normalized mRNA level of the Foxp3 gene increased by 80% (p <0.05) in 3-week diabetic rats (group 4) and by 3.1 times (p <0.05) in 5-week ESIDM rats, group 5 (Figure 2 C, D).

The mTOR is not only a central regulator of lipid metabolism that controls the processes of adipogenesis and lipolysis but also a regulator of the metabolism of the immune cells, which infiltrate adipose tissue [2]. The differentiation of individual subpopulations of T-cells depends on their expression of transcription factors [8] and the receptors of innate immunity [9, 10]. The level of progression of diabetes is largely influenced by the Treg cell subpopulation [11], the complexity and heterogeneity of which is confirmed by the detection of numerous tissue-specific Tregs, including the so-called VAT Tregs (visceral adipose tissue CD4+ Foxp3+ regulatory T cells) or “Fat Tregs” [12, 13]. Such VAT Tregs are characterized by a number of unique properties:

1. Foxp3+-expressing VAT Tregs account for 4060% of all CD4+ T-cells of the AT, which is much higher compared to their percentage in spleens and lymph nodes (5-20%) or non-lymphoid tissues (e.g., lungs and liver), as well as in subcutaneous fat [14];
2. VAT Tregs are characterized by a higher transcriptional level of the cytokine IL-10 (almost 136 times higher than its expression in regular Tregs). In turn, IL-10, which is produced by VAT Tregs, inhibits the production of inflammatory cytokines and chemokines induced by TNFα, increases GLUT4 expression in adipocytes, and restores AT sensitivity to insulin;
3. VAT Tregs have a higher expression of chemokines and chemokine receptors such as CCR1, CCR2, CCR9, CCL6, CXCL2 and CXCL10, and lower expression patterns of CCL5 and CXCR3 [15];
4. nuclear PPAR-γ receptors (peroxisome proliferator-activated receptor-gamma), which are deleted in regular Treg cells, are essential in VAT Treg for their functioning and accumulation in AT, as their specific deletion in Treg cells prevents the accumulation of these cells in AT, but not in lymphoid organs [16];
5. VAT Tregs express a specific T-cell receptor (TCR) repertoire that is different from the TCR repertoire of lymph node-associated Tregs.

The dependence of Treg differentiation on the level of mTOR expression has been shown by several research groups, including ours [17, 18]. Naive CD4+ cells differentiate into effector pro-inflammatory subpopulations of Th1, Th2, and Th17 cells, as well as cytotoxic CD8+ cells, and are activated at high mTOR activity [19]. Conversely, if mTOR activity in CD4+ cells is low, they differentiate into Treg cells that block the development of insulitis and the progression of diabetes [20].

The obtained results showed that metformin administration, which is capable of mTOR inhibition through AMP-activated protein kinase (AMPK), leads to an increase in the transcriptional activity of the Foxp3 gene in Treg cells of PPAT. Similarly, Shin N. et al. demonstrated that metformin increases the number and percentage of VAT Treg cells in mice that were on a high-fat diet [21]. However, it is unknown whether this effect of metformin is exclusively seen on VAT Treg and what molecular mechanisms mediate this effect [22]. In several animal studies, it was also shown that the administration or induction of Foxp3+ Treg cells led to profound reductions in autoimmune disease severity in models of diabetes, asthma, multiple sclerosis [23].

Some studies have shown a correlation between dysfunction of AT and the development of inflammatory and autoimmune diseases. Thus, Koenen et al. have shown that visceral AT has a more pronounced pro-inflammatory potential compared with subcutaneous AT [24]. In particular, the percentage of CD8+ -T lymphocytes in visceral AT was significantly higher than in subcutaneous AT (41.6 vs. 30.4%, p<0.05). Adipocytes of visceral AT were characterized by higher production of IL-1β (by 10 times, p <0.05), IL-18 (by 3 times, p <0.05), IL-6 and IL-8 (by 3 and 4 times, respectively) (p <0.05) compared to subcutaneous AT. Finally, caspase-1 activity in adipocytes of visceral AT was three times higher, which produces the conditions for activation of the inflammasome and explains the higher “susceptibility” of visceral AT to the development of inflammation [24].

Peripancreatic adipocytes (PPA) exert both systemic and paracrine effects on the pancreatic function. In mice with adipocyte-specific deletion of SUMO-specific protease (small ubiquitin-like modifier) SENP1, symptoms of type 1 diabetes were consequently developed. In a recent study conducted by Shao L. et al. in 2016, it was shown that the PPA of such SENP1-deficient mice increased the production of pro-inflammatory cytokines IL-6, TNFα and IL-1β compared to other fat depots [4]. Moreover, these cytokines not only do have a direct cytotoxic effect on pancreatic islets, but they also increase the expression of CCL5 and other chemokines (CCL2, CCL21, CXCL9, and CXCL10) in neighboring islets, which causes persistent inflammation in the pancreas through the involvement of effector Th1 and Th17 cells and reduction of Treg cells number. SUMO-specific protease can posttranslationally bind to cellular proteins and modulate their biological functions. The authors suggest that the SUMO-modulation of the NEMO protein (NF-kappa-B essential modulator) enhances the activity of NF-kB (nuclear factor kappa-light-chain-enhancer of activated B cells) and influences NF-kB-dependent production of insulitis-triggering cytokines [25]. Thus, PPAs can play an essential role in the progression of diabetes, providing a new understanding of the molecular pathogenesis of type 1 diabetes mellitus.

## Conclusions

The development of ESIDM causes transcriptional activation of the mTOR protein kinase gene in PPAT cells, does not affect the mRNA expression of the regulator of Treg cells differentiation, Foxp3, at the third week of pathological process development, but reduces it by 4.6 times (p <0.05) in 5-week diabetic rats. These changes are accompanied by an increase in the level of mRNA expression of the pro-inflammatory cytokines IL1β and IL17A in PPAT cells.

Metformin introduction to ESIDM rats inhibits mTOR mRNA expression by 4.5-5.9 times (p <0.05), resulting in an increase in the transcriptional activity of the Foxp3 gene in PPAT Treg cells by 80% (p <0.05) in 3-week and 3.1-fold (p <0.05) in 5-week ESIDM rats.

## Conflict of Interest

The authors confirm that there are no conflicts of interest.

## References

[1] Putilin DA, Kamyshnyi AM (2016). Сhanges of Glut1, mTOR and AMPK1α Gene Expression in Pancreatic Lymph Node Lymphocytes of Rats with Experimental Diabetes Mellitus. Medical Immunology.

[2] Ferrante AW (2013). The immune cells in adipose tissue. Diabetes Obes Metab.

[3] Kohlgruber AC, LaMarche NM, Lynch L (2016). Adipose tissue at the nexus of systemic and cellular immunometabolism. Semin Immunol.

[4] Shao L, Feng B, Zhang Y (2016). The role of adipose-derived inflammatory cytokines in type 1 diabetes. Adipocyte.

[5] Cai H, Dong LQ, Liu F (2016). Recent advances in adipose mTOR signaling and function: therapeutic prospects. Trends Pharmacol Sci.

[6] Lamming DW, Sabatini DM (2013). A Central role for mTOR in lipid homeostasis. Cell Metab.

[7] Kamyshnyi AM, Putilin DA, Konovalova OO, Kamyshna VA (2014). Особливості Peculiarities of TLR2 and TLR4 expression by adipocytes of parapancreatic tissue in experimental diabetes mellitus. Biomedical and biosocial anthropology.

[8] Koval HD, Chopyak VV, Kamyshnyi OM, Kurpisz MK (2018). Transcription regulatory factor expression in T-helper cell differentiation pathway in eutopic endometrial tissue samples of women with endometriosis associated with infertility. Cent Eur J Immunol.

[9] Topol I, Kamyshny A (2013). Study of expression of TLR2, TLR4 and transckription factor NF-kB structures of galt of rats in the conditions of the chronic social stress and modulation of structure of intestinal microflora. Georgian Med News.

[10] Degen AS, Koval GD, Sukhomlinova IE, Morozova OV, Kamyshnyi AM (2019). Analysis of cytoarchitectonics of TLR2+ AND TLR4+ lymphocytes and transcriptional activity of the genes Gp2, Spi-B, Nf-kB1, с-REL, TNFα and TNFr in galt of rats in experimental diabetes mellitus and after pentoxifylline administration. Medical Immunology.

[11] Suri-Payer E, Fritzsching B (2006). Regulatory T cells in experimental autoimmune disease. Springer Seminars in Immunopathology.

[12] Zeng H (2013). mTORC1 couples’ immune signals and metabolic programming to establish Treg-cell function. Nature.

[13] Chen X, Wu Y, Wang L (2013). Fat-resident Tregs: an emerging guard protecting from obesity-associated metabolic disorders. Obes Rev.

[14] Feuerer M, Herrero L, Cipolletta D (2009). Lean, but not obese, fat is enriched for a unique population of regulatory T cells that affect metabolic parameters. Nat Med.

[15] Cipolletta D, Kolodin D, Benoist C Tissular T(regs): a unique population of adipose-tissue-resident Foxp3+CD4+ T cells that impacts organismal metabolism. Semin Immunol.

[16] Cipolletta D, Feuerer M, Li A PPAR-gamma is a major driver of the accumulation and phenotype of adipose tissue Treg cells. Nature.

[17] Waickman AT, Powell JD (2012). mTOR, metabolism, and the regulation of T-cell differentiation and function. J. Immunol Rev..

[18] Chapman NM, Chi H (2014). mTOR signaling, tregs and immune modulation //. Immunotherapy.

[19] Spence A, Tang Q (2016). Restoring Regulatory T Cells in Type 1 Diabetes. Curr Diab Rep.

[20] Powell JD, Pollizzi KN, Heikamp EB (2012). Regulation of immune responses by mTOR. Annu Rev Immunol.

[21] Shin NR, Lee JC, Lee HY. (2014). An increase in the Akkermansia spp. population induced by metformin treatment improves glucose homeostasis in diet-induced obese mice. Gut.

[22] Cipolletta D (2014). Adipose tissue-resident regulatory T cells: phenotypic specialization, functions and therapeutic potential. Immunology.

[23] Zhou X, Tang J, Cao H (2015). Tissue resident regulatory T cells: novel therapeutic targets for human disease. Cell Mol Immunol.

[24] Koenen T, Stienstra R, van Tits L (2011). The inflammasome and caspase-1 activation: a new mechanism underlying increased inflammatory activity in human visceral adipose tissue. J. Endocrinology.

[25] Shao L. (2015). SENP1-mediated NEMO deSUMOylation in adipocytes limits inflammatory responses and type-1 diabetes progression. Nature communications.

